# Durable oxygen evolution reaction of one dimensional spinel CoFe_2_O_4_ nanofibers fabricated by electrospinning

**DOI:** 10.1039/c7ra11330g

**Published:** 2018-01-31

**Authors:** Zhengmei Zhang, Jingyan Zhang, Tao Wang, Zhiwei Li, Guijin Yang, Haiqin Bian, Jinyun Li, Daqiang Gao

**Affiliations:** Key Laboratory of Atomic and Molecular Physics, Function Material of Gansu Province, Northwest Normal University Lanzhou 730070 People's Republic of China zhangzhengmei@nwnu.edu.cn +86 09317971503; Key Laboratory for Magnetism and Magnetic Materials of Ministry of Education, Lanzhou University Lanzhou 730000 People's Republic of China gaodq@lzu.edu.cn +86 09318912234

## Abstract

One dimensional spinel CoFe_2_O_4_ nanofibers were synthesized *via* the electrospinning technique. The nanofibers were calcined at different temperatures. All CoFe_2_O_4_ nanofibers show excellent oxygen evolution reaction (OER) performance. The nanofibers calcined at 750 °C have a multi-particle nanochain structure. The nanochain exhibits excellent catalytic performance for OER in 1 M KOH (pH = 14) producing a current density of 10 mA cm^−2^ at an overpotential of 0.34 V, and the small onset potential of 1.32 V *versus* RHE, better than that of the commercial Ir/C (20%) catalyst. Furthermore, the stability of CoFe_2_O_4_ multi-particle nanochains toward the OER decreases by only 0.78% even after a long period of 80 000 s. Our finding suggests that CoFe_2_O_4_ nanofibers with a multi-particle nanochain structure could serve as a new group of OER electrocatalysts with excellent performance.

## Abstract 2

One dimensional spinel CoFe_2_O_4_ nanofibers were synthesized *via* the electrospinning technique.

## Graphical abstract

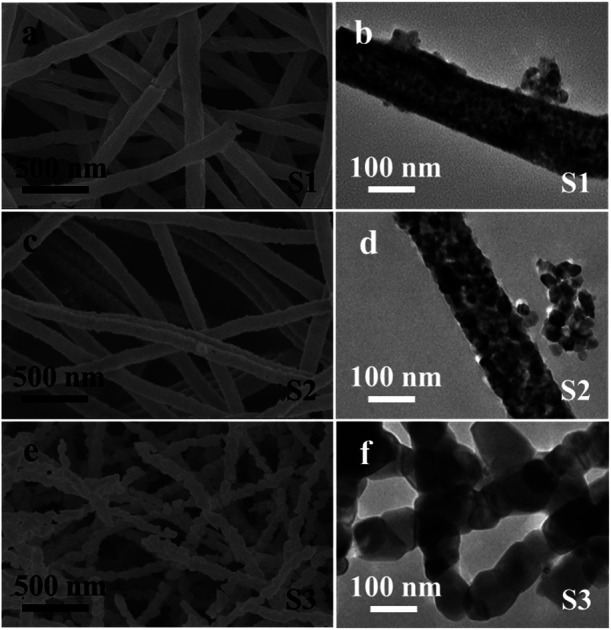

## Introduction

Hydrogen is considered to be an efficient, promising secondary, and environmentally benign energy resource compared to other fossil fuels.^1–6^ Among various hydrogen production methods, splitting water into hydrogen has stimulated considerable research interests in recent years, because it provides a green and sustainable approach.^7,8^ In the process of water-splitting, the hydrogen evolution reaction (HER) and oxygen evolution reaction (OER) are dramatically crucial for its gross efficiency.^9–11^ The OER is kinetically slow and requires the use of an electrocatalyst for expediting the reaction rate.^12–15^ Usually, precious metals such as Ru, Ir, and theirs oxides are utilized as commercial catalysts for the OER, but their scarce reserves and high cost impede their widespread application.^16–20^ Hence, it is extremely important to develop new OER catalysts with both high activity and low cost composed of easily obtained materials.^21,22^

Owing to their earth abundant nature, environmental benignity, and distinctive electrocatalytic activity for OER, spinel oxides have been discovered as good OER catalyst candidates.^23^ Co_3_O_4_,^24^ NiCo_2_O_4_,^11,24,25^ and CuMn_2_O_4_ (ref. 26) have been demonstrated as a group of efficient electrocatalysts for OER. Among different spinels, ferrite represents one of the most intriguing composite oxides due to their high abundance, high coercivity, moderate saturation magnetization, low-cost, low toxicity, good mechanical hardness, and rich redox chemistry.^27–29^ It has been broadly used in magnetic memory, sensors, drug delivery, photocatalytic, and anode materials for lithium ion battery. Particularly, cobalt ferrite can be a promising electrocatalyst due to its unique properties.^28^ The OER property could be optimized by reducing the dimension of electrocatalyst, in order to offer large specific surface area and enhance intimate contact with support electrodes.^12^ Cobalt ferrite nanoparticles are excellent electrocatalyst due to their large specific surface area. Unfortunately, the large active surfaces of granular nanocatalysts also lead to aggregation of material. One-dimensional nanofibers can effectively avoid the aggregation phenomenon and simultaneously offer large specific surface area.

Electrospinning represents an economical and promising synthetic technique to prepare one-dimensional nanofibers with different structural morphologies, including nanocables,^29^ porous nanotubes,^30^ nanotubes,^31^ nanoribbons,^32^ and nanorods.^33^ In this work, we focus on the CoFe_2_O_4_ nanofibers as a precursor which were fabricated using an electrospinning technique. The as-fabricated one-dimensional CoFe_2_O_4_ nanofibers exhibit benign electrocatalytic activity. The possible causes for the significantly enhanced OER performance of CoFe_2_O_4_ nanofibers are investigated in detail, which is correlated to morphology, specific surface area, and charge transfer ability, *etc.* Moreover, nanoscale characterization and OER property have been investigated. Our results provide a new way for devising the mass production of various one-dimensional ferrite materials, which is meaningful in providing an outstanding candidate for highly efficient and economical electrocatalysts.

## Experimental section

CoFe_2_O_4_ nanofibers were prepared by electrospinning. In a typical synthetic route, a polymer solution was first prepared by dissolving 0.784 g poly vinylpyrrolidone (PVP) into a mixed solution. The mixed solution contain ethanol, *N*,*N*-dimethyl formamide (DMF), iron nitrate nonahydrate (Fe(NO_3_)_3_·9H_2_O) and cobalt nitrate hexahydrate (Co(NO_3_)_2_·6H_2_O). For the typical synthesis, 2 mmol Fe(NO_3_)_3_·9H_2_O and 1 mmol Co(NO_3_)_2_·6H_2_O were initially dissolved in a mixed solution of 5.0 mL ethanol and 5.0 mL DMF under rapid stirring at room temperature by a magnetic stirrer to obtain a homogeneous sol–gel. The electrospinning process was carried out by applying a DC voltage of 14 kV, with a 14 cm spacing between the needle tip and the aluminum foil at room temperature. After the electrospinning process, the PVP/[Co(NO_3_)_2_ + Fe(NO_3_)_3_] precursor nanofibers were deposited on the aluminum foil. The as-spun nanofibers were then dried for 4 h in a drying oven at 60 °C. The nanofibers were then divided into three parts. These three parts were denoted as sample S1, S2, and S3, and calcined in furnace under air atmosphere at 550 °C, 650 °C, 750 °C for 2 h with the heating rate of 1 °C min^−1^, respectively. After all, the samples were naturally cooled down to room temperature.

The morphology and chemistry characterization of the CoFe_2_O_4_ nanofibers were performed using a field emission scanning electron microscope (FESEM, Hitachi S-4800) and a high resolution transmission electron microscope (HRTEM, FEI Tecnai F30) equipped with energy dispersive X-ray spectroscopy (EDX, Oxford Instrument). X-ray diffraction was used to study the crystal structure of the samples (XRD, X'Pert PRO PHILIPS with Cu Kα radiation, *λ* = 1.54056 Å). The chemical states of the elements were identified by the X-ray photoelectron spectroscopy (XPS, Kratos Axis Ultra).

The electrocatalytic activity for OER was measured using an electrochemical analyzer (CHI 660E, USA) in a configuration of conventional three-electrode cell, where the modified glassy carbon coated with catalyst was used as the working electrode, a Pt/C and a Ag/AgCl foil served as the reference and counter electrode, respectively. Linear sweep voltammetry (LSV) was performed in 1 M KOH at the room temperature with the scan rate of 2 mV s^−1^. The electrolyte was first cleaned by high purity N_2_ gas. The potentials were converted with reference to the reversible hydrogen electrode (RHE) through RHE calibration in 1 M KOH (pH = 14), *E*(RHE) = *E*(Ag/AgCl) + 197 mV + 0.059 × pH. The Nyquist plots were tested at 1.4 V *versus* RHE, and the impedance data was fitted to a simplified Randles circuit with frequency ranging from 100 kHz to 0.1 Hz to obtain the charge-transfer resistances.

## Results and discussions

### The morphological analysis

**Fig. 1:**
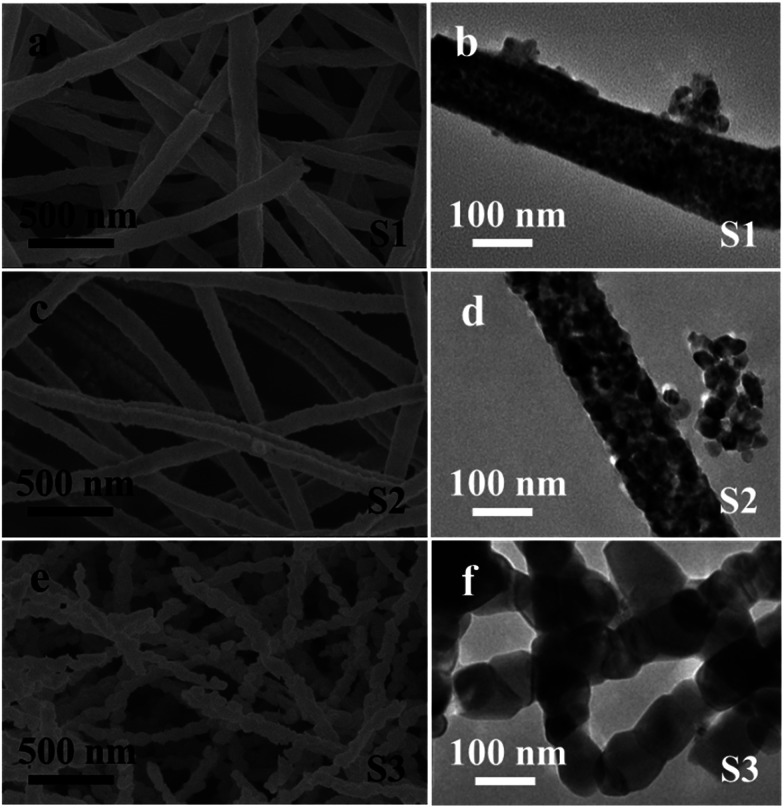
Morphology of CoFe_2_O_4_ nanofibers; (a, c, e) SEM images; (b, d, f) TEM images.

The role of calcination temperature on the morphologies and sizes of the CoFe_*x*_O_*y*_ nanofibers is investigated by varying the calcination temperatures at 550 °C, 650 °C and 750 °C. Fig. 1 shows the representative SEM, and TEM images of the CoFe_*x*_O_*y*_ nanofibers (S1–S3). Clearly, by the SEM images (Fig. 1a, c and e), all the samples present a column-like structure. Each nanofiber is continuous on structure and virtually uniform on particles sizes after the calcination process with length of several micrometers. The large-magnification TEM images of the nanofibers selected from S1–S3 further confirm the aforementioned column-like structure, as shown in Fig. 1b, d and f. It is clear that each nanofiber is consisted of abundant stacking nanoparticles. However, some remarkable changes are also noticed: the surfaces morphology of S1–S3 become rough because of the crystallization and growth of CoFe_2_O_4_ nanoparticles during calcination progress; the average diameters are estimated to be about 156 ± 13, 129 ± 16 and 92 ± 30 nm for S1, S2 and S3 in sequence, indicating that their diameters decrease with the increase of calcination temperature; nanofibers diameters slightly reduced but the particle sizes of samples increase rapidly when the calcination temperature goes up to 750 °C for S3, as shown in Fig. 1e and f, and the inset of Fig. 1f. Indeed, the S3 sample appears like multi-particles nanochain structure instead of nanofiber. EDX spectrum analysis of samples indicates that the atomic ratio of Co : Fe is 1 : 2, inferring that CoFe_2_O_4_ nanofibers have been synthesized under our experimental conditions.

### XRD analysis

**Fig. 2:**
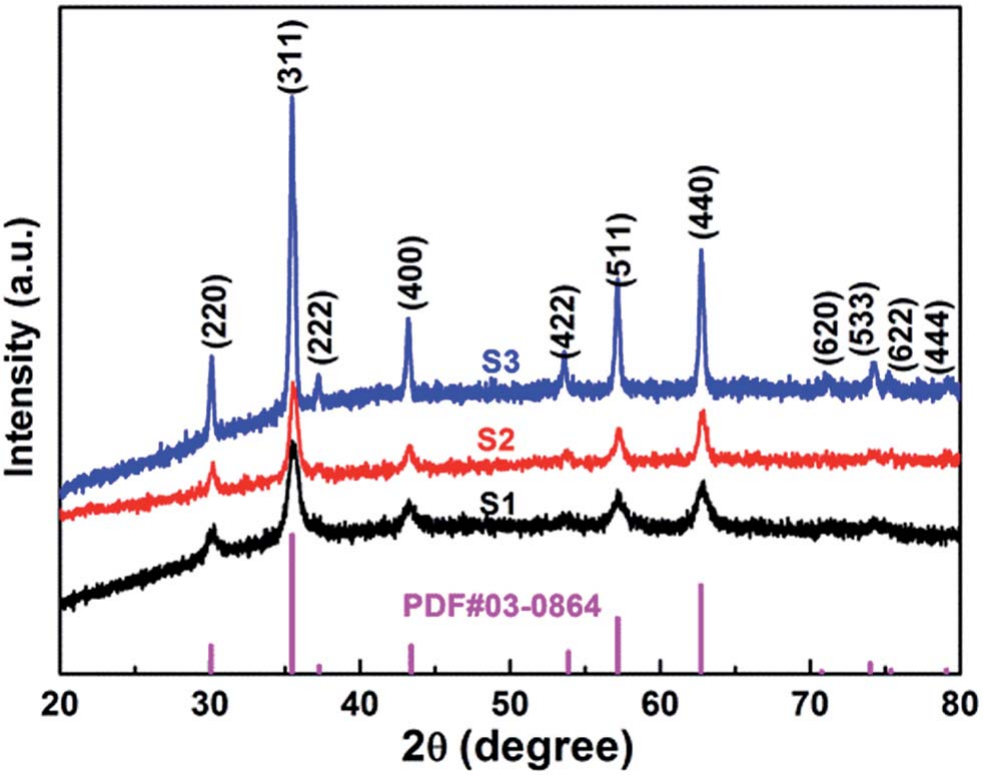
XRD patterns of CoFe_2_O_4_ nanofibers.

XRD analysis of the as-prepared CoFe_2_O_4_ nanofibers was performed, as shown in Fig. 2. It suggests that the S1–S3 are well crystallized with a cubic spinel structure. The peaks of samples can be indexed by (220), (311), (222), (400), (422), (511), (440), (620), (533), (622), and (444) lattice planes. No other impurity phases are detected, indicating that the formed CoFe_2_O_4_ nanofibers are all of the single spinel phase. Compared with S3, the diffraction peaks of S1 and S2 are obviously broadened, and the intensity of the diffraction peaks are less. It indicates the intensity of the diffraction peak gradually increases, and the peak width decreases with increasing calcination temperature. The strong and sharp XRD peaks confirm the excellent crystallization of the samples. The mean crystal sizes of the CoFe_2_O_4_ in the nanofibers are determined from the most intense (311) diffraction peak of the XRD line broadening using the Scherrer formula:1
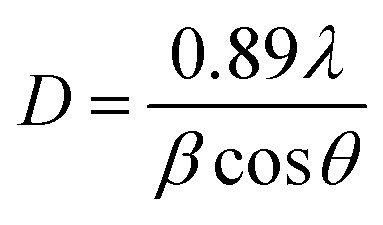
where, *λ* is the wavelength of X-ray source, *β* is the line broadening at half of the maximum intensity, and *θ* is the Bragg angle. The nanofibers are formed with CoFe_2_O_4_ nanoparticles, with uniform sizes. The average grain sizes for S1, S2 and S3 calculated by eqn (1) are about 10 nm, 15 nm and 27 nm, respectively. This result reveals that the grain size of CoFe_2_O_4_ is increased with increasing of calcination temperatures from 550 to 750 °C.

### XPS analysis

**Fig. 3:**
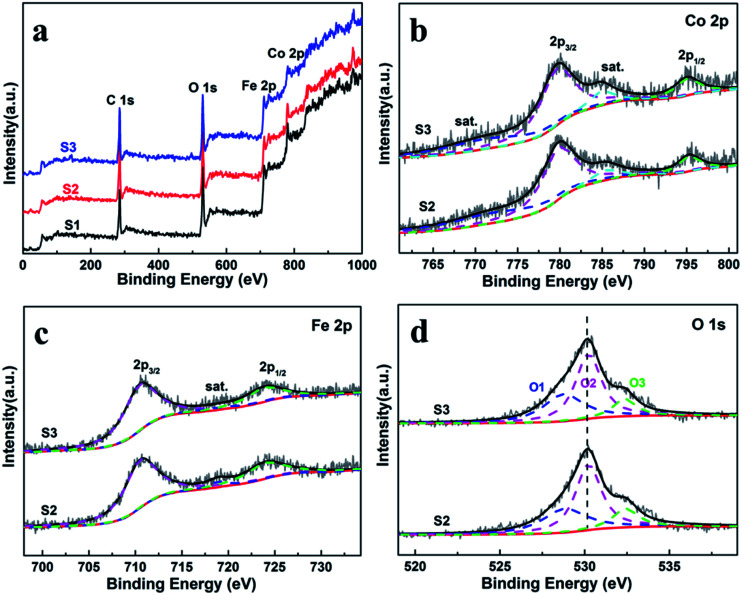
(a) XPS survey spectra of the CoFe_2_O_4_ nanofibers. XPS spectra for S2 and S3 (b) Co 2p, (c) Fe 2p, and (d) O 1s spectra.

**Fig. 4:**
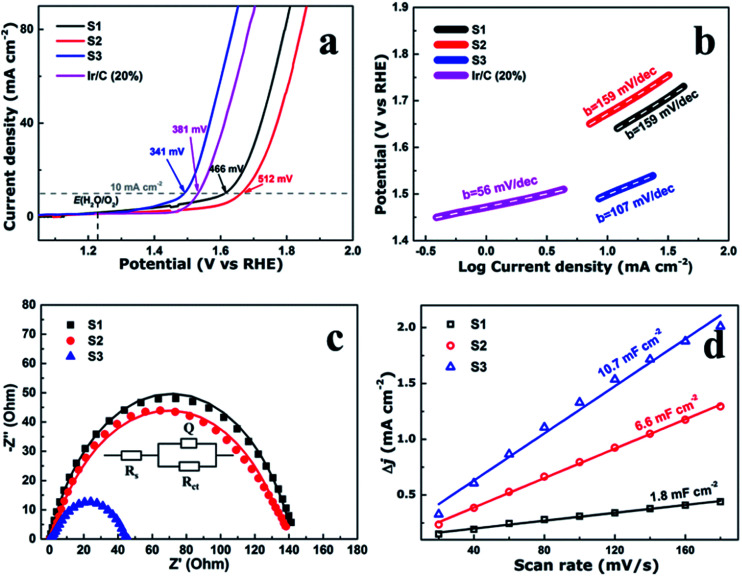
(a) Linear scan voltammograms (LSV), (b) corresponding Tafel plots, (c) Nyquist plots. *Z*′ is real impedance and *Z*′′ is imaginary impedance, and (d) the capacitive current density as a function of scan rate of the CoFe_2_O_4_ nanofibers.

X-ray photoelectron spectroscopy (XPS) were measured to obtain the valence state and chemical composition in CoFe_2_O_4_ nanofibers. After standardized by the C 1s peak (284.6 eV), as shown in Fig. 3a, C 1s, Co 2p, Fe 2p and O 1s are observed in the full spectrum of three samples. The high-resolution Fe 2p and Co 2p peaks can effectively examine the feature of metal ion species in the spinel oxide. The asymmetric Co 2p peaks in S2 and S3 can be further fitted (Fig. 3b). The main peaks at 780.0 eV (Co 2p_3/2_) and 795.3 eV (Co 2p_1/2_) are observed for CoFe_2_O_4_, with a spin orbit separation of 15.3 eV. In addition, there are two shake-up satellite peaks at around 771.3 eV and 784.8 eV at the lower binding energy edge of the Co 2p_1/2_ and Co 2p_3/2_ peaks. The shake-up satellite peaks and main peaks indicate the valence of Co is 2+ state.^33–35^ The Fe 2p spectrum (Fig. 4c) show two peaks at binding energies of around 710.7 eV and 724.1 eV, corresponding to Fe 2p_3/2_ and Fe 2p_1/2_, respectively. The peak at 718.9 eV is assigned to a satellite peak. Two main peaks with a spin–orbit separation of 13.4 eV are attributed to Fe 2p_3/2_ and Fe 2p_1/2_, indicating Fe is in the 3+ state.^33–35^ In Fig. 3d, the O 1s spectrum show three oxygen contributions, denoted as O1 (528.9 eV), O2 (530.2 eV) and O3 (532.5 eV), corresponding to the three different O in S2 and S3. The peak at 528.9 eV is due to metal–oxygen bonding,^36^ the peak at 530.2 eV is ascribed to a large number of defect sites with low oxygen coordination,^37^ and the peak at 532.5 eV is associated with hydroxyl group on the surface-adsorbed water molecules.^38^ Meanwhile, it can be observed that the core level spectra have no significant change with the increase of the calcination temperatures.

### The oxygen evolution reaction analysis

To evaluate the electrocatalytic performance of the CoFe_2_O_4_ nanofibers, the samples were first coated on a clean glass carbon electrode, which also provided the contact area for measurement (0.071 cm^2^ geometrical area). The mass loading of the uniform catalyst film was measured to be 0.32 mg cm^−2^. Linear sweep voltammetry (LSV) was performed in O_2_-saturated 1 M KOH with a pH of 14 at the room temperature. Fig. 4a shows the LSV curves of all the samples which have been normalized with an ohmic resistance (*IR*) correction. The *IR* losses caused by electrolyte resistance, were corrected by subtracting *IR*, and the value of *R* is low and consistent. Clearly, CoFe_2_O_4_ nanofibers exhibit considerably enhanced activity to OER with the smallest onset potential of 1.32 V *versus* RHE. The CoFe_2_O_4_ nanofibers even show a comparable onset potential with the conventional precious metal benchmark for OER (Ir/C, 1.47 V,^11^ and IrO_2_/C, 1.52 V (ref. 15)). In addition, the overpotential^39^ of S3 measured at current density of 10 mA cm^−2^, is 341 mV, which is lower than those of S1 (466 mV), S2 (512 mV) and even Ir/C (381 mV).^11^ Our results confirm that the S3 own a higher electrocatalytic activity for OER. Next, the OER electrocatalytic kinetics of the above catalysts were investigated by Tafel plots, as displayed in Fig. 4b. The Tafel slope of S3 is identified as 107 mV dec^−1^, which is the smallest among the CoFe_2_O_4_ nanofibers catalysts. It is noted that, despite with a similar structure, the S3 have a further decrease in the slope value compared with S1 and S2. This suggests that the S3 have the fast charge transfer progress.

To further measure the electrocatalytic efficiency in terms of OER for CoFe_2_O_4_ nanofibers, we also tested the Nyquist electrochemical impedance spectroscopy (EIS) to study the electrode kinetics under OER condition. The result of EIS is used to investigate the charge transfer resistance of the samples during the OER process, as shown in Fig. 4c. The equivalent circuit used to fit the EIS date is shown in the inset of Fig. 4c. *R*_ct_ values of 142.5, 140.3, and 44.8 ohm for S1, S2, and S3 suggest that an obvious decrease of charge transfer resistance is related to the improved OER performance. The finding suggests that S3 has the fastest charge transfer process and consequently facile OER kinetics at the electrode/electrolyte interfaces, and results in outstanding OER activity. In addition, the double-layer capacitances (*C*_d1_) were estimated of the CoFe_2_O_4_ nanofibers by using a cyclic voltammetry method. *C*_d1_, which is linearly proportional to the effective electrochemically active surface areas (ECSA), can be measured to compare the effective ECSA of the electrocatalysts. As shown in Fig. 4d, *C*_d1_ values are measured 1.8, 6.6, and 10.7 mF cm^−2^ for samples S1, S2, and S3, respectively. The highest *C*_d1_ of S3 is about 6 times of S1, indicating that sample S3 has the largest active sites.

**Fig. 5:**
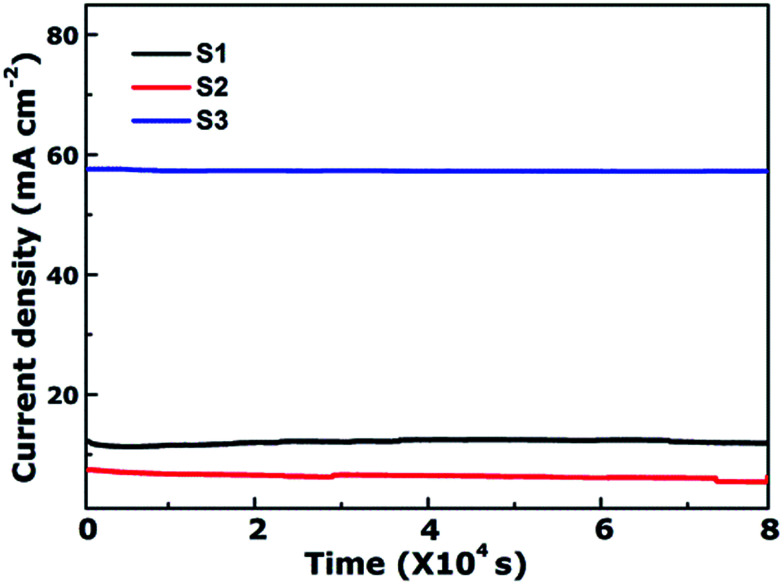
Time-dependent current density curves of CoFe_2_O_4_ nanofibers at 1.6 V *versus* RHE.

As is well-known, the stability of electrocatalysts is another critical parameter for practical utilization. To assess the durability study of the CoFe_2_O_4_ nanofibers for OER in alkaline electrolyte (pH = 14), continuous OER was performed at the samples' static overpotential. As shown in Fig. 5, the current density for S3 at 1.60 V *versus* RHE negligibly decreases by only 0.78% even after 80 000 s. In contrast, the OER current density on S1 and S2 decrease 2.87% and 23.88% after 80 000 s, respectively. Results reveal that S3 possess superior operational stability for future applications.

From the above results and discussions, the as-prepared CoFe_2_O_4_ multi-particles nanochains can be applied as an efficient OER electrocatalyst. The improved OER catalytic activity of S3 can be attributed to several reasons: (1) S3 has the best charge transfer rate owing to the lowest Tafel slope and charge transfer resistance, which is confirmed by Tafel slope and EIS measurements. (2) Rough surface and small diameters of S3 are favorable to increase the specific surface area of the sample and lead to more active sites between the electrode and electrolyte. It can be seen from the *C*_d1_ estimation. (3) The chemical synergistic effect of oxygen, such as more oxygen vacancies, also contributes to the enhanced activity, but it can be ignored owing to same XPS's results of three samples.

## Conclusions

In summary, CoFe_2_O_4_ nanofibers have been synthesized by electrospinning technology followed *via* calcination under different temperature. The nanofibers were characterized by SEM, TEM, EDX, XRD, and electrochemical measurement in detail. Our results showed that increasing calcination temperature caused the morphology change of the CoFe_2_O_4_ nanofibers. Further, we show that the nanofiber calcined at 750 °C has a multi-particles nanochain structure, and act as an efficient OER catalyst. CoFe_2_O_4_ multi-particles nanochains exhibit enhanced OER catalytic activity compared with CoFe_2_O_4_ nanorods, with lower onset potential and overpotential. Notably, the stability of S3 shows negligible degradation in OER current density over 80 000 s of continuous operation. This work offers a new pathway for designing high efficient OER electrocatalysts using a simple electrospinning technique.

## Conflicts of interest

There are no conflicts to declare.

## Supplementary Material
